# Integrating simulation training during clinical practice in nursing homes: an experimental study of nursing students’ knowledge acquisition, self-efficacy and learning needs

**DOI:** 10.1186/s12912-022-00824-2

**Published:** 2022-02-22

**Authors:** Camilla Olaussen, Simen A. Steindal, Lars-Petter Jelsness-Jørgensen, Ingunn Aase, Hege Vistven Stenseth, Christine Raaen Tvedt

**Affiliations:** 1 Lovisenberg Diaconal University College, Lovisenberggata 15b, 0456 Oslo, Norway; 2 University of Stavanger, Postboks 8699 Forus, 4036 Stavanger, Norway; 3 Department of Health and Social Studies, Østfold University College, Fredrikstad, Norway; 4 Østfold Hospital Trust, Kalnes, Norway; 5 SHARE- Centre for Resilience in Healthcare, Faculty of Health Sciences, University of Stavanger, Stavanger, Norway

**Keywords:** Clinical learning, Knowledge, Nursing education, Nursing homes, Self-efficacy, Simulation training

## Abstract

**Background:**

Limited access to supervision, feedback and quality learning experiences pose challenges to learning in the clinical setting for first-year nursing students who are beginning their clinical experiences. Prior studies have indicated that simulation training, as a partial replacement of clinical practice hours, may improve learning. However, there has been little research on simulation training integrated as a partial replacement during first-year students’ clinical practice in nursing homes. The primary aim of this study was to examine first-year nursing students’ knowledge acquisition and self-efficacy in integrating a partial replacement of clinical hours in nursing homes with simulation training. Its secondary aim was to examine perceptions of how learning needs were met in the simulated environment compared with the clinical environment.

**Design:**

The primary aim was addressed using an experimental design that included pre- and post-tests. The secondary aim was investigated using a descriptive survey-based comparison.

**Methods:**

First-year students at a Norwegian university college (*n* = 116) were asked to participate. Those who agreed (*n* = 103) were randomly assigned to the intervention group (*n* = 52) or the control group (*n* = 51). A knowledge test, the General Self-efficacy Scale and the Clinical Learning Environment Comparison Survey were used to measure students’ outcomes and perceptions. The data were analysed using independent samples *t*-tests, chi-square tests and paired samples *t*-tests.

**Results:**

Knowledge scores from pre- to post-tests were significantly higher in the intervention group than in the control group with a medium to large effect size (*p <* 0.01*, Hedges’ g =* 0.6). No significant differences in self-efficacy were identified. Significant differences (*p* <  0.05) were observed between the simulated and the clinical environment with regard to meeting learning needs; effect sizes ranged from small and medium to large (Cohen’s d from 0.3 to 1.0).

**Conclusion:**

Integrating the partial replacement of clinical hours in nursing homes with simulation training for first-year nursing students was positively associated with knowledge acquisition and meeting learning needs. These findings are promising with regard to simulation as a viable partial replacement for traditional clinical practice in nursing homes to improve learning.

## Background

The Norwegian Coordination Reform, which was introduced in January 2012 [1], has resulted in the transferral of patients suffering serious, complex and treatment-intensive conditions to nursing homes, thus placing extensive demands on staffing and competence [2]. In addition, nursing homes often struggle with nurse shortages due to recruitment difficulties and high turnover [3, 4]. Nursing education programmes are essential in meeting increasingly complex care needs and demands, recruiting and retaining nurses in bedside positions and ensuring future patient safety and quality in nursing homes [5].

Supervised experiences with patients in real clinical settings are an important part of nursing students’ clinical education [6, 7]. According to the traditional Norwegian clinical education model for first-year nursing students, all hours of clinical practice are conducted in nursing homes and supervised by onsite registered nurses [8]. Nursing students need feedback, guidance and support to acquire the knowledge of managing challenging learning situations in clinical practice and to build competency for self-efficacy and safe patient care [9, 10]. However, students’ access to supervision, feedback and quality learning experiences is not always optimal [11, 12]. In nursing homes, the limited number of registered nurses that serve as supervisors can pose a significant challenge to the learning of first-year students who are just beginning their clinical experiences [13–15]. Prior studies have indicated that integrating simulation training as a partial replacement of clinical practice hours may improve learning [16, 17].

When used as preparation for clinical practice, studies have reported that simulation training has positive effects on student outcomes such as knowledge, decision-making, self-confidence and self-efficacy [18–20]. In an umbrella systematic review, Cant and Cooper [19] found that simulation training statistically improved self-efficacy in pre- and post-test studies, and in experimental designs self-efficacy was superior to that of other teaching methods. Further they found that many reviews agreed on outcomes of knowledge, although no overall quantitative effect was derived [19]. In a randomized controlled trial comparing students’ knowledge and self-confidence scores before and after attending simulation training, Haddeland et al. [20] identified significantly greater improvement in the intervention group compared with the control group. A systematic review and meta-analysis on the effectiveness of simulation training based on life-threatening clinical condition scenarios found no significant effect on students’ self-confidence and self-efficacy but demonstrated that simulation training is superior to other teaching methods in improving knowledge and performance [21]. However, there are few previous studies on the partial replacement of clinical hours by simulation training among first-year students in nursing homes. The National Council of State Board Nursing’s (NCSBN) National Simulation Study was a two-year longitudinal, randomised controlled study in which clinical hours were replaced by 25 and 50% simulation training in two intervention groups, respectively. These intervention groups were then compared with a control group that had up to a 10% replacement. No statistically significant differences between the groups were found [22]. However, the NCSBN study showed a possible advantage of partial replacement for the development of clinical competency in the medical-surgical and community health areas, but a potential disadvantage in the perinatal, paediatric, and mental health areas [22, 23]. Curl et al. [24] used a quasi-experimental design and found that students who replaced 50% of clinical practice in obstetrics, paediatrics and mental health had similar or better results with regard to knowledge than those who had undergone traditional clinical practice. A systematic review found that replacing clinical hours by simulation training had no significant impact on student outcomes, such as knowledge acquisition and self-confidence, compared with traditional clinical practice [23]. A meta-narrative review by Roberts et al. [25] found no significant differences in student outcomes but highlighted that the lack of clearly stated number of hours of simulation versus number of clinical hours meant the generalisability of research findings was difficult.

Roberts et al. [25] reported the need for continued research to determine the possible advantages or disadvantages of simulation training as a partial replacement for clinical hours. Davis et al. [26] emphasised that it is essential to determine the optimal combination of simulation and clinical hours. Larue et al. [23] called for studies to examine various simulation–clinical combinations, depending on the clinical context to which students are exposed. Simulation training as a partial replacement during clinical practice in nursing homes for first-year students is a combination of simulation and clinical training that has not yet been well studied.

In the current study, we examined knowledge acquisition and self-efficacy among first-year nursing students who received a 10.7% partial replacement of clinical hours in nursing homes with simulation training (the intervention group) and first-year nursing students who received the traditional Norwegian education model with clinical studies limited to nursing homes (the control group). As a secondary aim, we examined how well learning needs were met in the clinical environment compared with the simulated environment among the students in the intervention group.

## Methods

### Design

The primary aim was addressed using an experimental design that included pre- and post-test comparisons of students’ knowledge and self-efficacy in the intervention group (the combination of simulation–clinical training) versus the control group (only clinical training). The secondary aim was addressed using a descriptive survey-based comparison in the intervention group.

### Participants and setting

The study was conducted at a Norwegian university college that provides nursing education at the bachelor level. One class of first-year nursing students (*N* = 116) who were enrolled in the second semester of their bachelor education during the spring of 2020 were asked to participate. Those who agreed (*n* = 103) were randomly assigned to either the intervention group (*n* = 52) or the control group (*n* = 51). Randomisation was performed by the university administration staff using the random *between* function in Microsoft Excel to avoid selection bias. Three students from the control group left the education programme before the initial pre-test, which resulted in a control group of 48 students and a total of 100 participants. Before the practice placement period commenced, the university administration staff ensured that the students in the intervention group were placed in nursing homes that were different from those assigned to the control group. None of the 13 nursing homes involved offered simulation training for students during the practice period.

#### The control group: “traditional clinical practice”

The control group attended a seven-week practice period of 224 h in nursing homes, which is hereafter referred to as “traditional clinical practice”*.*

#### The intervention group: “clinical practice with simulation as partial replacement”

The intervention group attended a seven-week practice period of 224 h in nursing homes, of which 24 h (10.7%) were replaced by simulation training on three separate days during the practice period, which is hereafter referred to as “clinical practice with simulation as partial replacement”.

### Description of the intervention: clinical practice with simulation as partial replacement

The simulation training was scheduled in weeks 2, 4 and 6 of the seven-week practice period. The INACSL Standards of Best Practice: Simulation© and the National League for Nursing/Jeffries simulation theory, which provide systematic steps for designing quality simulation experiences, guided the design of the simulation training [27, 28]. The scenarios used in the simulation training were designed to resemble situations that students were likely to encounter in their nursing home practice. To enhance the level of fidelity in the scenarios, high technology full-body mannequins (NursingAnne®; Laerdal™) with vital signs that reflected the patient’s diagnosis were used, the patient’s environment was designed to replicate a nursing home, and the students could immerse themselves in the simulation experiences as autonomous clinicians making their own decisions and demonstrating their knowledge [27, 29, 30]. The patient scenarios are presented in Table 1.

**Table 1:** Scenarios and objectives of the simulations in clinical practice with simulation as partial replacement

Scenarios:	Situation presented for the students:	Objectives presented for the students:
1.Nursing home patient with chronic pulmonary disease deterioration	Nursing home patient, female, 75 years old, sufferers from chronicle obstructive pulmonary disease (COPD), uses salbutamol 2 mg × 4 administered by inhalation. The patient is anxious. Her skin is warm and sweaty.	Perform relevant clinical observations and measure vital signs Identify the patient’s problems, needs and possible complications Make clinical decisions, prioritize actions based on vital sign assessments, knowledge and trained skills Evaluate effect of actions Contact, inform and collaborate with physician
2. Nursing home patient dementia, developing delirium caused by urinary retention	Nursing home patient, male, 89 years old with a mild degree of dementia, and a chronic urinary retention. Permanent catheter, and a urine sample for bacteriological cultivation are ordinated. The patient’s behaviour has changed, with a deteriorating confusion. The patient has been given Stesolid 2 mg without effect.	
3. Administration of medications to nursing home patient with left ventricular heart failure	Nursing home patient, male, 75 years old, sufferers from a left ventricular heart failure. The patient uses heart medications, and is scheduled for his intramuscular injection with B12 depot 1 mg. The patient is not cooperating, seems to struggle with his breath while lying down. He does not want his medication.	

Previous research has suggested a 2:1 clinical-to-simulation ratio (i.e., two clinical hours count as 1 h of simulation training) because of the intensity and efficiency of simulation training compared with the clinical setting [11, 16, 24]. Because of the resources available in this study, the university administration gave permission to replace 3 days (24 h, 10.7%) of the 28 days (224 h) in “traditional clinical practice”. Each day was replaced by the following: i) time for students to prepare for the simulation training individually by reading preparation materials before the simulation training commenced (1 h); ii) the simulation training which included three steps: initial briefing, the active simulation, and debriefing (3 h with a 2:1 simulation ratio = 6 h); and iii) time for the students to write individual reflection notes after the simulation training was completed (1 h). Preparation materials with information about logistics, meeting times, specific scenarios, and learning objectives were provided before each simulation training and were accessible by students in their learning management systems.

The intervention group attended the simulation training in six groups of eight to ten students each. Each simulation training started in step 1, the initial briefing (30–45 min), that offered an overview of the environment, objectives and technical equipment [31]. In step 2, three to four students participated as nurses in active simulations (30–40 min), while the other students held the role of active observers. The students switched roles during the simulation training days, which allowed all students to practice as nurses. In step 3, the scenarios were deconstructed and analysed in a facilitated debriefing that lasted a minimum of 90 min. The Promoting Excellence and Reflective Learning in Simulation (PEARLS) framework was used to guide the debriefing in four distinctive phases: the reaction, the description, the analysis and the summary phase [32]. Two experienced facilitators employed at the university college (i.e., the fifth author and an additional teacher) conducted the briefing, the active simulation and the debriefing, while the simulation operators regulated the technical features of the simulator and presented the patients’ voices.

### Data collection

To achieve the primary aim, data were collected using a multiple-choice knowledge test and the General Self-efficacy Scale (GSE). The Clinical Learning Environment Comparison Survey (CLECS) was used to achieve the secondary aim. The participants completed all questionnaires electronically.

#### Data collection: primary aim

The data collected at different time points related to the primary aim are presented in Table 2. Pre- and post-tests were completed 1 week prior to and 1 week following the clinical practice, respectively.

**Table 2:** Data collection for the primary aim of the study

Participants Spring 2020	Pre-test Before the practice period in January 2020	The practice period of 7 weeks	Post-test After the practice period in March 2020
Intervention group	Knowledge test General Self-Efficacy Scale	Clinical practice with simulation (simulation performed in week 2, 4 and 6	Knowledge test General Self-Efficacy Scale
Control group	Knowledge test General Self-Efficacy Scale	Traditional clinical practice	Knowledge test General Self-Efficacy Scale

##### Knowledge test

The knowledge test was specifically designed for the present study, as no appropriate tests were identified in the literature. The multiple-choice test contained 30 questions on the areas of respiration, circulation, elimination and drug handling. The test was developed based on the students’ curriculum and expected learning outcomes during clinical practice in the nursing homes. Four response alternatives in addition to “I don’t know” were given. Only correct answers were given one point, and higher scores were indicative of better learning outcomes (scores ranged from 0 to 30 points). The facilitators in the simulation training were blinded to the content of the knowledge test to reduce method bias that could affect the intervention group’s test outcomes.

A panel of experts comprising four teachers responsible for first-year education courses was consulted to ensure the content validity of the test [33]. In addition, the test was administrated to four second-year nursing students who were asked to evaluate the structure, meaning of the questions, wording and test instructions [34]. The last step was to pilot the final test version in a group of 15 s-year nursing students to detect potential flaws and weaknesses and to estimate a provisional standard deviation (SD) for the power analysis.

##### The GSE (Norwegian version)

The GSE is a 10-item psychometric scale that is designed to assess optimistic self-beliefs to cope with difficult demands. The scale has been translated into Norwegian and validated [35]. The GSE uses a four-point scale that measures the respondents’ agreement with the statements (1 = Not at all true, 2 = Hardly true, 3 = Moderately true, 4 = Exactly true), with a score from 10 to 40 points. A high score represents a more optimistic assessment of general self-efficacy.

#### Data collection: secondary aim

##### CLECS (Norwegian version)

The CLECS was administered to the intervention group to examine the students’ perceptions of how well learning needs were met in the simulated versus the clinical environment 1 week following the clinical practice period. The CLECS is specifically designed for this purpose, and it has been psychometrically tested in Norwegian [36, 37]. Items are scored using a four-point Likert scale: 1 = “Not met”, 2 = “Partially met”, 3 = “Met”, 4 = “Well met”, in addition to “not applicable”. For each item, the students selected a score for both the clinical and the simulated environment [37]. The results were provided as mean scores for the clinical environment and for the simulated environment in six subscales: Communication (four items); Nursing Process (six items); Holism (six items); Critical Thinking (two items); Self-Efficacy (four items); and the Teaching–Learning Dyad (five items).

### Variables

The variables used to address the primary aim of the study were the pre- and post-knowledge and self-efficacy mean scores from both groups. The variables used to address the secondary aim of the study were the intervention group’s mean scores on the six subscales in the CLECS for both the clinical environment and the simulated environment.

### Data analyses

A power analysis with a provisional SD of 3.9 estimated from the pilot testing of the knowledge test showed that a sample size of 27 students in each group was sufficient to identify a difference in improvement of 3 points, with a maximum risk of a type 1 error of 5% (*p* <  0.05) and a strength of 80%. Descriptive statistics are presented as means and SD for continuous variables and as frequencies and proportions for the categorical variables. Differences in demographic variables, self-efficacy and knowledge scores between the groups were analysed using independent sample *t*-tests (two-tailed) and chi-square tests. Differences in self-efficacy and knowledge scores within the groups and differences in how well learning needs were met in the clinical environment compared with the simulated environment were detected by paired sample *t*-tests (two-tailed). Hedges’ g was used to calculate the effect sizes for the independent sample *t*-tests (by dividing the mean difference between the groups by the pooled SD with weights for the sample sizes). Cohen’s d was used for the paired sample *t*-tests (by dividing mean differences by the SD of the difference). Cohen’s [38] operational definitions of small (= 0.2), medium (= 0.5) and large effects (= 0.8) were used.

The significance level was set at 5%, *p* <  0.05. IBM SPSS Statistics version 26 (IBM, Armonk, NY, USA) was used to conduct the analyses.

### Ethical considerations

The study was approved by the Norwegian Social Science Data Services (ref. 875,320) and performed in accordance with the 2013 revised version of the Declaration of Helsinki. Participation was voluntary and based on written informed consent. It had no consequences for the students’ educational progression. Students could withdraw at any point during the study.

## Results

None of the study participants had prior experience in simulation training. The pre-test was completed by 97 of 100 (97%) students, of whom 52 were assigned to the intervention group (53.6%) and 45 were assigned to the control group (46.3%). There were no significant differences in demographic variables, baseline knowledge or self-efficacy scores between the groups (data not shown). The post-test was completed by 88 of these 97 students (90.7%), whereas 50 students were in the intervention group (57%) and 38 students were in the control group (43%). There were no statistically significant differences in the demographic variables, baseline knowledge or self-efficacy scores between the control group (*n* = 38) and the intervention group (*n* = 50) for those who completed both the pre- and post-tests (Table 3).

**Table 3:** Demographic variables and pre-test results of study participants who completed both pre- and post-tests knowledge and self-efficacy

	Control *n* = 38	Intervention *n* = 50	*p*
Age: mean (SD)	22.9 (4.4)	23.3 (5.7)	0.7
Female: *n* = (%)	32 (84.2)	45.0 (90.0)	0.4
Years working in health care as nursing assistants or healthcare assistants: mean (SD)	1.4 (2.1)	2.0 (2.1)	0.2
Former higher education in other professions or areas: *n* = (%):			
1. No former higher education	30 (78.9)	43 (86.0)	0.4
2. Former bachelor/master’s degree	8 (21.1)	7 (14.0)	
Pre-test knowledge	11.8 (3.9)	13.2 (4.1)	0.1
Pre-test self-efficacy	29.1 (3.8)	28.2 (4.6)	0.3

*n* number of participants, *SD* standard deviation, *p p*-value

The dropout rate for the control group was 20.8, and 3.8% for the intervention group.

### Knowledge and self-efficacy

Differences in knowledge scores from pre- to post-test within the control group and the intervention group were statistically significant, while differences in self-efficacy scores from pre- to post-test within the groups were not (Table 4). There were statistically significant differences in the post-test knowledge scores between the intervention group and the control group (mean difference 3.6, 95% Cl 2.1–5.0, *p* < 0.01, Hedges’ g = 0.9)*.* However, no statistically significant differences in post-test self-efficacy scores were observed between the groups (mean difference 1.4, 95% Cl − 0.9 – 3.0, *p* = 0.1, Hedges’ g = 0.1).

**Table 4:** Comparisons of pre- and post-test scores within the control and intervention groups and mean improvement comparisons between the groups

Control *n* = 38							Intervention *n* = 50						Mean improvement comparison between groups^a^	
	Pre-test	Post-test	Mean diff.	95% CI		*p*	Pre-test	Post-test	Mean diff.	95% CI		*p*	*p*	g_Hedges_
				Lower	Upper					Lower	Upper			
Knowledge (Mean (SD))	11.8 (3.9)	14.0 (4.1)	2.2 (3.5)	1.0	3.3	0.001	13.2 (4.1)	17.6 (3.6)	4.4 (3.8)	3.3	5.5	< 0.001	< 0.01	0.6
Self-efficacy (Mean (SD))	29.1 (3.8)	30.3 (3.5)	1.2 (4.0)	−0.1	2.5	0.08	28.2 (4.6)	28.9 (3.9)	0.7 (3.8)	−0.4	1.8	0.2	0.6	0.1

*SD* standard deviation, *Mean diff* mean difference between pre- and posttest, *CI* confidence interval, *p p*-value; g_Hedges_ effect size

^a^Comparisons of mean difference from pre- to post- tests between the control and intervention groups

The mean improvement in knowledge scores from the pre-test to the post-test was higher in the intervention group than in the control group (Table 4). The difference in mean improvement between the groups was statistically significant (mean difference 2.2, 95% Cl 0.6–3.8) with a medium to large effect size (Table 4). No statistically significant difference in mean self-efficacy improvement between the pre-test and the post-test were observed in the control versus the intervention group (mean difference 0.5, 95% Cl − 1.2 – 2.1)*.* A small effect size was also observed (Table 4).

### Perceptions of how learning needs were met in the intervention group

Mean scores in the intervention group on how learning needs were met were significantly higher in the simulated learning environment than in the clinical environment on all six subscales of the CLECS. On three subscales (Nursing Process, Self-Efficacy and Teaching–Learning Dyad), the effect sizes were medium to large, while small effect sizes were observed in the remaining subscales (Table 5).

**Table 5:** The intervention group’s (*n* = 50) reports of how well learning needs were met in the clinical practice environment versus the simulated environment

Variables	Simulated environment	Clinical environment					
	Mean [SD]	Mean [SD]	Mean diff. (SD)	95% CI		*P*	d_Cohen_
				Lower	Upper		
Communication (4 items)	3.4 (0.5)	3.1 (0.6)	0.3 (0.7)	0.1	0.5	<.01	0.4
Nursing Process (6 items)	3.7 (0.3)	3.0 (0.6)	0.7 (0.7)	0.5	0.9	<.001	1.0
Holism (6 items)	3.0 (0.6)	2.8 (0.7)	0.2 (0.7)	0.0	0.4	.04	0.3
Critical Thinking (2 items)	3.6 (0.6)	3.3 (0.7)	0.3 (0.8)	0.1	0.5	<.01	0.4
Self-Efficacy (4 items)	3.4 (0.6)	3.0 (0.7)	0.4 (0.5)	0.2	0.5	<.001	0.7
Teaching–Learning Dyad (5 items)	3.8 (0.3)	3.1 (0.7)	0.8 (0.7)	0.6	1.0	<.001	0.9

*SD* standard deviation, *Mean diff* mean difference between clinical and simulated environment, *CI* confidence interval, *p p*-value, d_Cohen_ effect size

## Discussion

In this study, we demonstrated that first-year nursing students had higher knowledge acquisition when traditional clinical practice in nursing homes was partially replaced by simulation training. The effect size value indicated the practical significance (i.e., a difference large enough to be meaningful in real life) of this result [38]. However, we observed no significant difference in levels of general self-efficacy. The first-year students scored the simulated environment higher on meeting their learning needs compared with the clinical environment. The effect size values of this result indicated practical significance in the areas of Nursing Process, Self-Efficacy and Teaching–Learning Dyad [38].

Supportive guidance in linking theory to practice is vital in learning how to provide quality nursing care for patients [39]. However, the theoretical component of the nursing curriculum can be overwhelming for students [40]. Students in nursing home practice placements have reported little time for reflection and care reasoning with their supervisors [12, 41]. The supervision tends to be task-oriented and related to routine care, and transferable knowledge is not always recognised by students [42, 43]. Ironside et al. [10] found that students frequently missed cues indicating that the patient situations were more complex than merely completing assigned tasks. The current study found a significant positive difference in knowledge acquisition from clinical practice with simulation training as a partial replacement compared with traditional clinical practice in nursing homes. Supervision by teachers and the time available for reflection in the simulation training may have enhanced the students’ understanding of complex concepts and promoted the self-identification of gaps in knowledge, thus motivating students to further learning [42, 44]. Based on the design of the current study, we could not rule out that the requirement of preparing for the simulation training may have affected the results. However, nursing students are expected to be exposed to as well as process knowledge in preparing for clinical experiences in both traditional and simulated environments [45].

Although the factor of self-efficacy is widely believed to increase knowledge [46], we observed no significant difference in levels of general self-efficacy between the two groups. Shinnick and Woo [47] found no correlation between self-efficacy and knowledge in simulation training, nor was self-efficacy a predictor of “good” knowledge scores. In the current study, the general self-efficacy scores in both groups may have been related to the high grades required to be enrolled at the university college where the study was conducted. Prior success in school-related tasks may have contributed to the students’ already optimistic sense of general self-efficacy [48]. Moreover, the GSE may not have been sensitive or detailed enough to best reveal students’ levels of self-efficacy in managing the care of nursing home patients. An interesting finding in the current study was that the intervention group rated the simulation environment to meet learning needs related to self-efficacy significantly higher than the clinical environment. The reason for this result may have been that the self-efficacy statements in the CLECS pointed more directly to self-beliefs related to patient care compared with the general statements in the GSE. However, prior studies that have examined the impact of simulation training on general self-efficacy using the GSE have reported significant differences in general self-efficacy in the fields of psychiatric nursing, community healthcare nursing, communication and paediatrics [49–51].

An important step in improving nursing students’ clinical education by a partial replacement of clinical hours by simulation training is to understand how learning needs are met by the two methods. In the current study, the learning needs were rated to be better met in the simulated environment. The CLECS covers different aspects of students’ learning needs from the time they receive a patient through the evaluation of patient care [37]. We observed that the subscales Teaching–Learning Dyad and Nursing Process had the highest mean differences between the two learning environments. The Teaching–Learning Dyad was defined by Leighton [37] as the interactive relationship between supervisor/teacher and student in which both have shared responsibility for the learning outcomes, while Nursing Process was described as a systematic patient care approach that involves assessment, nursing diagnosis, planning, implementation and evaluation. It has been reported that students in clinical practice placements may experience a disconnection between the taught versus the observed nursing role, and that they may feel that they are left on their own to learn by trial and failure [12, 40]. An explanation for the difference related to the Teaching–Learning subscale may be that in simulation training, support and feedback from and collaboration with the teacher are inherent features [27]. Furthermore, the current study incorporated academic- and practice-focused simulation training that focused on nursing observations, assessments and evaluation of care, which may have enhanced students’ understanding of the nursing process as a structured approach to care, thereby influencing the scores.

### Implications for practice

The unmet learning needs of students should drive changes in traditional practice placement models [15, 37]. Our results provide evidence related to knowledge acquisition and meeting learning needs, which may justify the partial replacement of clinical hours in nursing homes by simulation training for first-year nursing students. In planning partial replacements of clinical hours, educators may be guided by tools such as the CLECS in their work to design simulation training that may compensate for learning needs that are not properly met in the clinical environment and thereby potentially negatively affect learning outcomes [36]. However, the number of clinical practice hours required in nursing education programmes is set by the relevant governing bodies; for example, the European Union directive specifies that 50% of the nursing education programme must be dedicated to clinical practice placements [52]. Thus, the replacement of clinical hours may demand changes in official clinical requirements. As partial replacement, simulation training also has resource implications [22]. In addition to a considerable amount of faculty time, scheduling issues and the availability of simulation facilities are challenges faced by educators in implementing simulation training as a partial replacement for traditional clinical practice [53].

### Strengths and limitations of the study

In the present study, differences in knowledge acquisition were measured by a multiple-choice test. Questions have been raised about the appropriateness of using multiple choice as a method of assessing the effectiveness of simulation experiences, as multiple-choice questions tend to assess lower levels of cognitive processing [54]. A multiple-choice test may not be the best tool to evaluate the potential higher order thinking benefits of clinical education. In the present study, we could not control for the participants’ different experiences in clinical practice, nor could we control the distribution of participants between private and municipal nursing homes, which could potentially have influenced the results.

The loss of participants to follow up was higher in the control group than in the intervention group. The control group participants might have felt less obligated to complete the study because they did not receive anything beyond the traditional clinical practice. Nevertheless, no statistically significant differences in demographics and pre-test scores between the groups that completed both pre- and post-tests were observed. Although the sample size was adequate to support the findings of this study, it could also be viewed as a limitation. The results were derived from a limited sample drawn from a single nursing education institution. Expanding the study to include other nursing education institutions would allow for the greater generalisability of the findings.

## Conclusion

The results of the present study showed that the partial replacement of hours of clinical practice in nursing homes by simulation training was positively associated with knowledge acquisition and meeting the learning needs of first-year nursing students. These findings are promising regarding simulation training as a viable partial replacement of traditional clinical practice in nursing homes to improve learning. Our findings may help educators to develop future clinical practice models as well as to inspire further necessary research on integrating simulation training as part of clinical practice placements.

## Data Availability

The datasets analysed during the current study are available from the corresponding author upon reasonable request.

## Abbreviations

NCSBN: The National Council of State Board Nursing
INACSL: International Nursing Association for Clinical Simulation and Learning
GSE: The General Self-Efficacy Scale
CLECS: The Clinical Learning Environment Comparison Survey
PEARLS: Promoting Excellence and Reflective Learning in Simulation
